# Sortase A Induces Th17-Mediated and Antibody-Independent Immunity to Heterologous Serotypes of Group A Streptococci

**DOI:** 10.1371/journal.pone.0107638

**Published:** 2014-09-18

**Authors:** Xin Fan, Xiaoshuang Wang, Ning Li, Honglian Cui, Baidong Hou, Bin Gao, Paul Patrick Cleary, Beinan Wang

**Affiliations:** 1 Key Laboratory of Pathogenic Microbiology and Immunology, Institute of Microbiology, Beijing, China; 2 Key Laboratory of Infection and Immunity, Institute of Biophysics, Chinese Academy of Sciences, Beijing, China; 3 Department of Microbiology, University of Minnesota, Minneapolis, Minnesota, United States of America; Quuen's University Belfast, United Kingdom

## Abstract

Group A streptococci (GAS) are associated with a variety of mucosal and invasive human infections. Recurrent infections by highly heterologous serotypes indicate that cross-serotype immunity is critical for prevention of GAS infections; however, mechanisms underlying serotype-independent protection are poorly understood. Here we report that intranasal vaccination of mice with Sortase A (SrtA), a conserved cell wall bound protein, reduced colonization of nasal-associated lymphoid tissue (NALT) by heterologous serotypes of GAS. Vaccination significantly increased CD4^+^ IL-17A^+^ cells in NALT and depletion of IL-17A by neutralizing antibody prevented GAS clearance from NALT which was dependent on immunization with SrtA. Vaccination also induced high levels of SrtA-specific antibodies; however, immunized, B cell-deficient mice cleared streptococcal challenges as efficiently as wild type mice, indicating that the cross-serotype protection is Th17-biased and antibody-independent. Furthermore, efficient GAS clearance from NALT was associated with a rapid neutrophil influx into NALT of immunized mice. These results suggest that serotype independent immune protection against GAS mucosal infection can be achieved by intranasal vaccination with SrtA and enhanced neutrophil function is critical for anti-GAS defense and might be a target for prevention of GAS infections.

## Introduction

The existence of more than 150 M-serotypes of group A streptococci (GAS) has been a major impediment to GAS vaccine development. This heterogeneity and the fact that the dominant serotypes at any point in time vary considerably in different regions of the world [1] question the feasibility of a multivalent M protein vaccine approach. The need for a vaccine is eminent, as GAS infections are associated with a variety of autoimmune sequelae, such as acute rheumatic fever, rheumatic heart disease, and acute glomerulonephritis [2], ultimately having a significant impact on human public health [3], [4].

Sortase A (SrtA), a transpeptidase produced by GAS and other Gram-positive bacteria, recognizes secreted proteins containing an LPXTG motif and covalently attaches these proteins to cell wall peptidoglycan [5]. Up to 12 GAS cell wall-anchored proteins contain LPXTG-like motifs [6], such as M proteins, protein F, C5a peptidase (ScpA), protein G-related α2-macroglobin-binding protein (GRAB), and pili [7]. Mutation of SrtA in most human Gram-positive pathogens, including GAS, dramatically reduced their ability to infect animals [8]–[13]. Analysis of 24 isolates of 12 GAS serotypes indicates that *SrtA* is present in all strains examined [14], indicating that SrtA is highly conserved and for that reason is a potential target for vaccine candidate.

We previously demonstrated that dominant CD4^+^ IL-17A^+^ cells in nasal-associated lymphoid tissue (NALT) are induced by GAS and are required for efficient clearance of bacterial colonization from NALT [15], [16]. Recent studies of the immune responses to *Streptococcus pneumoniae* and *Klebsiella pneumoniae* demonstrate that Th17 responses induced by mucosal immunization provide efficient serotype-independent protection [17], [18]. These findings suggest that Th17-based immunity may lead to successful cross-serotype protection against GAS. Here, we report that intranasal (i.n.) immunization by repeated infection or by inoculation with SrtA and cholera toxin B subunit (CTB) protected against infections by different serotypes of GAS. The results revealed that mucosal Th17 response and neutrophil activation play an important role in anti-GAS defense and suggest that exploiting these functions may provide new strategies for prevention of GAS diseases.

## Materials and Methods

### Ethics statement

This study was performed in strict accordance with the recommendations in the Guide for the Care and Use of Laboratory Animals of the IMCAS (Institute of Microbiology, Chinese Academy of Sciences) Ethics Committee. The protocol was approved by the Committee on the Ethics of Animal Experiments of IMCAS (Permit Number: PZIMCAS2011002). Mice were bred under specific pathogen-free conditions in the laboratory animal facility at IMCAS. All animal experiments were conducted under isoflurane anesthesia, and all efforts were made to minimize suffering.

### Cloning and expression of recombinant SrtA protein

The cDNA encoding SrtA was amplified by PCR from GAS M1 strain (90–226). SrtA was cloned into the Nde I and Xho I sites of the pET28 vector. BL21 cells containing pET28-SrtA were cultured in LB medium containing kanamycin 50 µg/ml at 37°C with shaking at 220 rpm until the OD_600_ reached 0.6. Then, isopropyl β-D-1-thiogalactopyranoside (IPTG) was added to a final concentration of 1 mM, and the BL21 cells were cultured overnight at 37°C. Cells were harvested by centrifugation, and the pellets were resuspended in 50 ml PBS containing extra salt (400 mM/ml NaCl). The mixture was sonicated until it became clear, and then centrifuged for 20 min at 12,000 rpm. Recombinant SrtA protein in the supernatant was purified by affinity chromatography on NiOS_2_-charged chelating Sepharose Fast Flow (GE Healthcare Bio-Sciences AB, Uppsala, Sweden) gel according to the manufacturer's instructions. The eluate of recombinant SrtA was concentrated and purified by Superdex 200 size exclusion chromatography using an AKTA purifier 2000 system (GE Healthcare Bio-Science AB) in 20 mM Tris (pH 7.4) and 50 mM NaCl. The recombinant SrtA preparation used for mouse immunization had a purity of >95%.

### Bacterial strains and culture conditions

The serotype M1 strain (90–226) of GAS was obtained from the University of Minnesota. Serotypes M28, M49, and M12 were clinical isolates kindly provided by Dr. X. Shen (Beijing Children's Hospital Beijing 100045, PR China). All strains were maintained on sheep blood agar and grown in THB-Neo (Todd-Hewitt broth supplemented with 2% Neopeptone). Overnight cultures (OD_560_∼1.1) were washed with and resuspended in PBS and used for all infections. CFUs were verified by plating on blood agar.

### Mice

Female C57BL/6 (B6) and BALB/c mice (aged 4 to 6 weeks) were purchased from Vital River Laboratory Animal Technology, whose colonies were all introduced from Charles River Laboratories. µMT mice were obtained from Dr. B. Hou, and their phenotypes were determined by surface staining of blood cells with anti-CD19 (6D5, Biolegend). Those mice were of C57BL/6 background, and control C57BL/6 mice were age and gender matched when used. C57BL/6 mice were used in adoptive transfer assays and BALB/c mice were used in other mouse experiments unless otherwise indicated.

### Animal immunization and infection

Mice were anesthetized with an isoflurane/oxygen mixture for 1 min and i.n. inoculated with the indicated immunogen in 10 µl PBS (5 µl per nostril). SrtA (20 µg) and cholera toxin B subunit (CTB) (1 µg) (Sigma, St Louis, MO) were used together or separately. For immunization with live GAS mice were inoculated i.n. with GAS M1 strain 0.5−1.0×10^8^/mouse. Control mice were given PBS alone. Mice were immunized at weekly intervals and challenged with sub-lethal doses of the required serotypes of GAS (2−8×10^8^/mouse) 10 days after the last immunization. 24 hr after challenge, NALT tissues were collected for CFU counting or FACS analysis as previously described [15]. Blood was taken by cardiac puncture, and oral wash samples were collected by rinsing the mouse oral cavity with 0.5 ml PBS. Serum and oral wash samples were stored at −20°C.

### RNA extraction and real time-PCR

Total RNA was isolated from NALT with an RNeasy mini kit (Qiagen) and reversed-transcribed with a High Capacity cDNA Transcription Kit (ABI) according to the manufacturer's instructions. Quantitative real time-PCR was performed on an ABI 7300 System (Applied Biosystems) using a SYBR Premix Ex Taq kit (Takara). Primers for real-time PCR were as follows: IL-17A forward, CGCAAAAGTGAGCTCCAGA; IL-17A reverse, TGAGCTTCCCAGATCACAGA; IFN-γ forward, GCGTCATTGAATCACACCTG; IFN-γ reverse, GAGCTCATTGAATGCTTGGC; and GAPDH forward, CATGGCCTTCCGTGTTCCTA; and GAPDH reverse: GCGGCACGTCAGATCCA.

The real time PCR data were analyzed by the delta delta CT analysis method [ΔΔCt  =  ΔCt (*Il17a*/*Ifnγ* in immunized - *Gapdh* in immunized) - ΔCt (*Il17a*/*Ifnγ* in PBS control - *Gapdh* in PBS control)] and the fold change of each sample was expressed as 2^-ΔΔCt^.

### Antibody measurement by ELISA

ELISA was used to measure specific antibodies in mouse sera and oral wash. Serial dilutions of each sample were dispensed on Maxisorp 96-well plates (Nalgene Nunc International) coated with recombinant SrtA at 0.3 µg/well. Antibody binding was detected by horseradish peroxidase-conjugated anti-mouse IgG or IgA (Southern Biotech), followed by 3, 3', 5, 5'-Tetramethylbenzidine staining (Sigma). Absorbance was measured at 450 nm (minus 630 nm for wavelength correction). Results are expressed as OD_450_ values.

### Intracellular staining and flow cytometry

NALT cells (1×10^6^ cells) were stimulated in the presence of 50 ng/ml PMA (Sigma) 500 ng/ml ionomycin (Sigma) in complete RPMI medium at 37°C in 5% CO_2_. After 30 min, 5 µl/ml Brafeldin A solution (Biolegend) was added, and the cells were incubated for another 4 hr. Cells were stained for surface markers with anti-CD4-FITC (GK1.5, BD bioscience) or anti-CD4-APC (GK1.5, Biolegend), anti-CD8-PerCP (53–6.7, Biolegend), anti-CD3-FITC (17A2, Biolegend), anti-CD49b-PE (DX5, Biolegend), anti-γδ TCR-PE-Cyanine7 (eBioGL3, eBioscience) and anti-CD44-APC (eBio1M7, eBioscience) as needed, and fixed in 4% paraformaldehyde (Solarbio technology) in PBS on ice. For intracellular staining, fixed cells were permeabilized and stained in saponin (0.1% in PBS; Sigma) with anti-IL-17A-PE and anti-IFN-γ-APC (BD bioscience), in the presence of 1.5% BSA. Samples were acquired on a FACSCalibur flow cytometer (BD Biosciences) and data were analyzed with FlowJo software (Tree Star). The frequency of each CD4^+^ T cell subpopulation was then expressed as the fraction of the overall cytokine response by CD4^+^ T cells (%). (We always found higher CD4^+^ IL-17^+^ cells in C57BL/6 than in BALB/c mice following the same treatment). Flow cytometry analysis for neutrophil and macrophage was performed as described in [19]. Briefly, neutrophils were initially gated by their characteristic forward (FSC) and side scatter (SSC) profiles covering both live and apoptotic neutrophils (medium to high SSC and FSC). Those cells were then analyzed for CD11b^+^ Gr-1^high^ cells, and cells that stained positively were quantified as a percentage of total gated cells.

### Neutralization of IL-17A in vivo

Mice were immunized as described above. To block IL-17A, immunized mice were injected with 100 µg IL-17 antibody (17F3, BioXCell) or isotype control (MOPC-21, BioXCell) every other day, starting the day after the last immunization. Mice were challenged with live GAS 7 days after the last immunization and euthanized 24 hr following the challenge to determine CFU in NALT [15].

### Enrichment and adoptive transfer of CD4^+^ T cells or CD19^+^ B cells

C57B/6 mice (4 to 6 weeks) were i.n. immunized with SrtA/CTB three times. 10 days after the last immunization, lymph nodes and the spleens were harvested and pooled. Single cell suspensions were made and CD4^+^ T cells were purified ( >93%) using anti-CD4 microbeads (Miltenyi Biotec) according to the manufacturer's protocol [15]. For purifying CD19^+^ B cells, single cell suspensions were sorted using a FACSAria sorter with FACSDiva software (BD Bioscience) after FITC-conjugated anti-CD19 (Biolegend) staining. A small volume from each sample was stained and analyzed by flow cytometry for the purity and phenotype of the cells. The purities of sorted CD4^+^ T cells and CD19^+^ B cells were 93% and 95%, respectively. Naïve female C57B/6 mice aged 4 to 6 weeks were used as recipients, and 1×10^7^ of either CD4^+^ T cells or CD19^+^ B cells were transferred to each mouse in a volume of 200 µl by tail vein injection. 24 hr later, recipient mice were challenged i.n. with 2×10^8^ CFUs of GAS M28 per mouse. All mice were killed 24 hr after the challenge; NALTs were harvested, and cell lysates were diluted and plated on sheep blood agar plates for CFU counts.

### Statistics

Statistical analyses were performed with two-tailed unpaired Mann-Whitney U nonparametric t tests using GraphPad Prism (Version 5.01 for Windows; GraphPad Software). The data were considered significantly different at *P*≤0.05.

## Results

### 1. I.n. immunization with M1 strain of GAS provided cross-serotype protection

**Figure 1:**
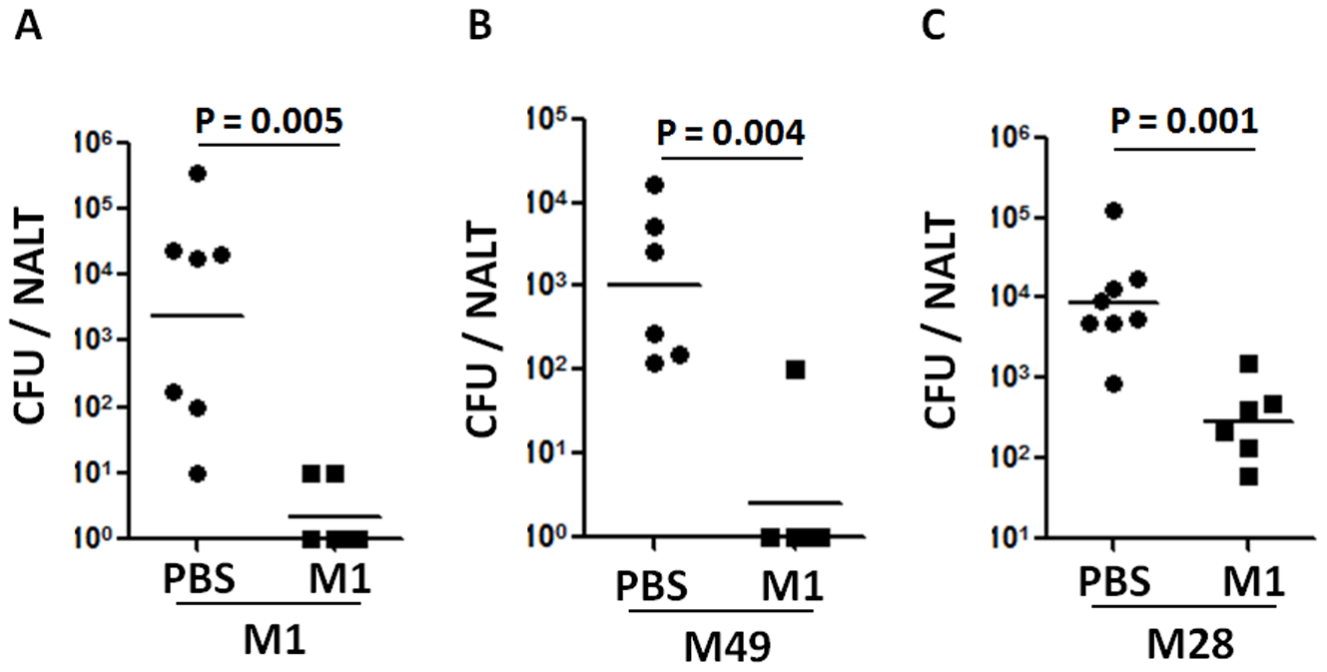
Immunization (i.n.) with live GAS strain M1 provided cross-serotype protection. Mice were i.n. immunized three times with live M1 strain (0.5−1.0×10^8^/mouse). 10 days after the last immunization mice were challenged with a sublethal dose of GAS serotypes M1 (*A*), M49 (*B*), or M28 (*C*) (Material and Methods). NALTs were taken 24 hr after challenge and homogenized for CFU counting. Data are geometric means of two independent experiments.

GAS have a tropism for mouse NALT [20]. We previously found that multiple i.n. immunization with live GAS induces predominant Th17 cell response in NALT of mice and promotes GAS clearance from NALT by 24 hr after challenge [15]. It is reported that Th17 responses mediate cross-serotype immunity to *Klebsiella pneumoniae* [18]. These findings led us to hypothesize that the immunity induced by i.n. immunization with GAS could be serotype-independent. This was tested with the mouse model of GAS mucosal infection [20]. Groups of mice were i.n. immunized with the M1 strain 90–226 three times and challenged with M1 (highly invasive), M49 (noninvasive) [21] or M28 streptococci (associated with puerperal sepsis and neonatal infections and the common cause of other invasive infections and pharyngitis in many countries) [22]. 24 hr after challenge viable bacteria associated with NALT were determined. CFU of all three serotypes were much lower in NALT from immunized mice than from PBS control mice (Fig. 1). This indicated that i.n. immunization with M1 induced protective immunity to heterologous serotypes and that the protection was independent of specific M protein epitopes.

### 2. I.n. immunization with SrtA protein and CTB promoted cross-serotype clearance of GAS from NALT

**Figure 2:**
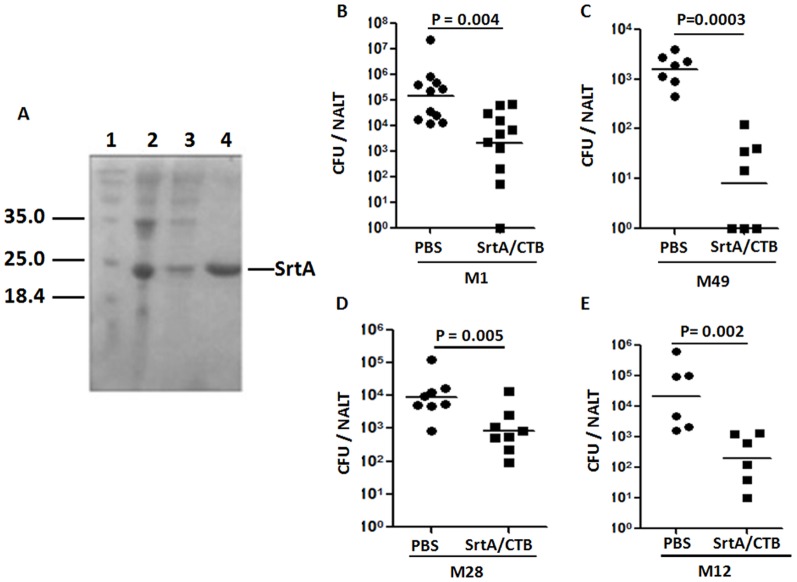
Immunization (i.n.) with SrtA/CTB promoted cross-serotype clearance of GAS. Recombinant SrtA protein in sonicated BL21 cells was purified by affinity chromatography on NiOS_2_-charged chelating Sepharose Fast Flow gel and analyzed by SDS-PAGE (*A*). 1. Molecular weight; 2. Whole cell lysate loaded on the column; 3. Unbound; 4. Eluted protein. Mice were i.n. immunized with SrtA and CTB three times. 10 days after the last immunization mice were challenged with a sublethal dose of live GAS serotypes M1 (*B*), M49 (*C*), M28 (*D*) or M12 (*E*). NALTs were taken 24 hr after challenge and homogenized for CFU counting. Data are geometric means of two to three independent experiments.

The cross-serotype immunity induced by i.n. immunization with GAS could be generated by highly conserved streptococcal proteins. The conserved nature of SrtA prompted us to test whether SrtA could provide protection against GAS mucosal colonization. Mice were i.n. immunized with recombinant SrtA protein cloned from the M1 90–226 strain (Fig. 2A, Materials and Methods) and cholera toxin B subunit (CTB) as a mucosal adjuvant. After three times of immunization at 1-week intervals mice were challenged with M1 strain 90–226 and CFUs were determined 24 hr after challenge. Significantly lower numbers of CFUs were found in immunized than in PBS control mice (Fig. 2B). To rule out a possible contribution of contaminated LPS in the recombinant SrtA, LPS in the samples was removed to <0.1 EU/µg SrtA protein. Comparison of the two preparations of SrtA in two independent animal protection experiments showed that mice immunized with LPS-cleaned SrtA reduced challenge GAS as well as those immunized with LPS-containing SrtA, indicating that LPS did not contribute to the protection (data not shown). To test if SrtA/CTB could provide cross-serotype immunity immunized mice were challenged separately with an M49, M28, or M12 strain (the predominant circulating type in the 2011 Hong Kong outbreak of scarlet fever) [23]. Compared with unimmunized mice, the number of viable streptococci in NALT of immunized mice was considerably decreased after challenge with each of the different serotypes (Fig. 2C, D, and E), indicating that vaccination with SrtA protein induced M type-independent clearance of streptococci from NALT.

### 3. Recall Th17 responses were induced by different serotypes of GAS in NALT of mice that were i.n. immunized with SrtA/CTB

**Figure 3:**
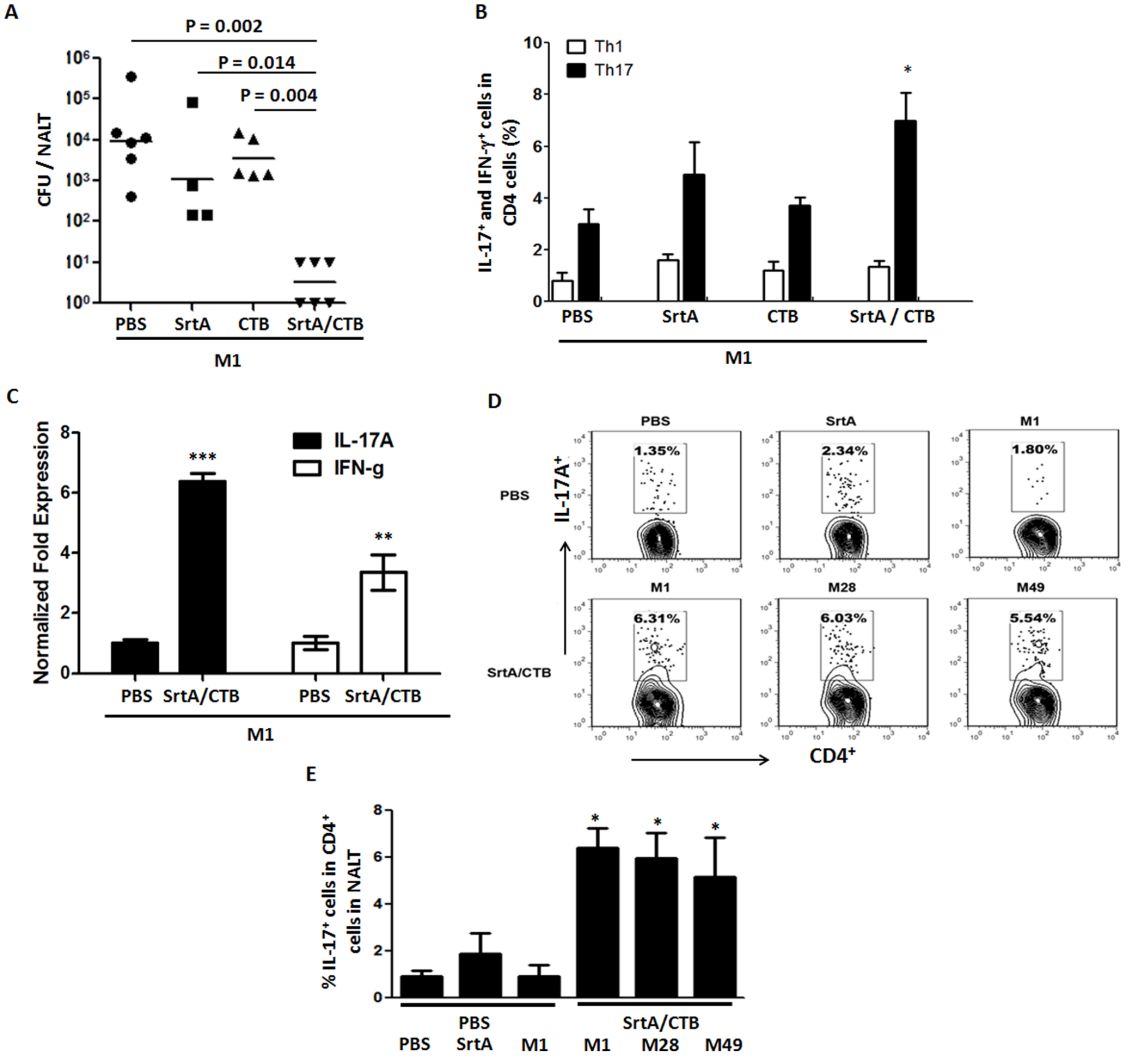
Specific recall Th17 responses were induced in SrtA/CTB-immunized mice by heterologous GAS strains. Mice were immunized i.n. three times with SrtA, or CTB, or SrtA and CTB, and challenged with live M1 strain 10 days after the last immunization. (*A*) 24 hr after challenge CFUs retained in NALT were determined. Data are geometric means of two independent experiments. (*B*) Mice were immunized and challenged as in (*A*). Three days after challenge, mice were euthanized and NALT cell suspensions were stained with FITC-CD4 followed by intracellular staining with PE-IL-17 and APC-IFN-γ and analyzed by FACS. Data are means ± SEM of three mice per group. * *P*≤0.05. (*C*) NALTs from immunized (SrtA/CTB) and PBS control mice were taken one day after challenge. Total RNA was isolated from NALTs and *Il17a* and *Ifnγ* m-RNA levels were determined by RT-PCR. Data were expressed as 2^-ΔΔCt^ (means ± SEM of three mice per group, ** *P*≤0.01, *** *P*≤0.001). (*D*) Mice were immunized as in Fig. 2 and 10 days after the last immunization each group of mice were challenged with strain M1, M28 or M49. Control mice received PBS, SrtA or live M1 strain. Three days after challenge NALTs were taken and NALT cells were stained and analyzed by flow cytometry as in (*B*). (*E*) Quantification of CD4^+^ IL-17^+^ T cells as in (*D*). Data are presented as the means ± SEM (n = 3) from two independent experiments. * *P*≤0.05 (compared to PBS/PBS group).

**Figure 4:**
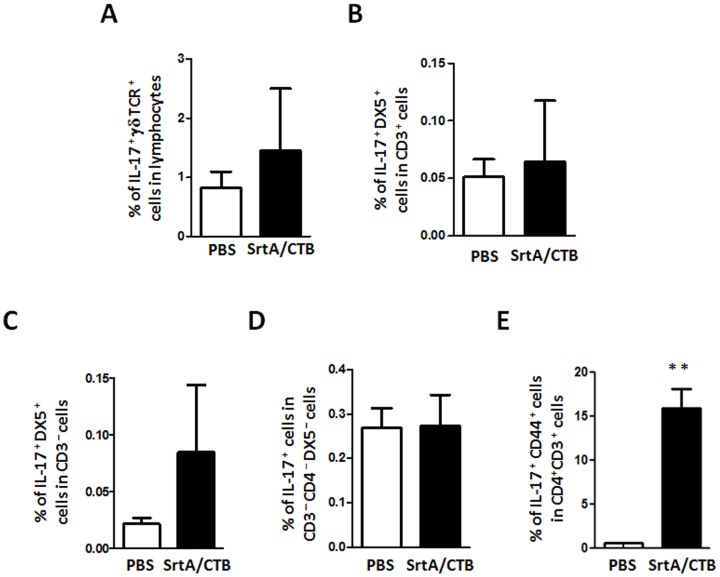
IL-17-producing CD4^+^ cells were the predominant responsive cell population in SrtA/CTB-immunized mice after GAS challenge. Mice were immunized and challenged as in Fig. 3. Five days after challenge, immunized (SrtA/CTB) and PBS control mice were euthanized and NALT cell suspensions were stained with antibodies for surface markers (CD4, CD3, DX5, and γδ TCR) as needed followed by intracellular staining with PE-IL-17 and analyzed by FACS. (*A*) IL-17^+^ γδ TCR^+^ (γδ T) cells. (*B*) IL-17^+^ DX5^+^ CD3^+^ (NKT) cells. (*C*) IL-17^+^ DX5^+^ CD3^–^ (NK) cells. (*D*) IL-17^+^ CD3^–^ CD4^–^ DX5^–^ (ILC3) cells. (*E*) IL-17^+^ CD3^+^ CD4^+^ CD44^+^ (memory Th17) cells. Data are means ± SEM of three mice per group. ** *P*≤0.01.

We sought to determine if efficient clearance of GAS induced by SrtA/CTB is correlated to Th17 activation. CTB is known to augment Th17 responses [24]; therefore, the requirement of CTB for the protection was tested. Mice were immunized with SrtA or CTB alone or SrtA combined with CTB and then challenged with the M1 strain. CFU in the homogenized NALT was significantly lower in mice immunized with SrtA/CTB than in those immunized with either SrtA or CTB only, or PBS (Fig. 3A), indicating that CTB adjuvanicity is required for induction of protective immunity. T cell analyses showed that the number of CD4^+^ IFN-γ^+^ cells did not change in any immunized group; whereas, CD4^+^ IL-17^+^ cells increased following SrtA/CTB immunization, compared to naïve controls (Fig. 3B) and were inversely proportional to residual CFUs in NALT. CD4^+^ IL-17^+^ cells remained at relatively low levels when mice were immunized with SrtA or CTB alone. RT-PCR analyses demonstrated that *Il17a* mRNA from NALT was dramatically increased in immunized mice and *Ifnγ* mRNA levels were also increased but to a lower degree (Fig. 3C) one day after challenge, confirming that the IL-17 cytokine response is predominant in NALT of immunized mice. CD4^+^ IL-17^+^ cells were also analyzed after mice were challenged with M28 or M49 strain. As expected, these cells increased in response to inoculation with each serotype (Fig. 3D, lower panel and 3E, column 5–6), but did not in naive mice (PBS control) when they were challenged with SrtA or viable M1 strain (Fig. 3D, upper panel and 3E, column 2 and 3). IL-17A is also produced by many innate immune cells, such as γδ T, NKT, NK, and group 3 innate lymphoid cells (ILC3). To confirm that the IL-17A-producing cells are T helper cells, NALT cells were analyzed by cell type specific markers. IL-17A-producing γδ^+^ T (γδTCR^+^), NKT (CD3^+^ DX5^+^), NK (CD3^−^ DX5^+^), and ILC3 (CD3^−^ CD4^−^ DX5^−^ ) cells were small populations in NALT (Fig. 4). Numbers of those cells did not change significantly in immunized and PBS control mice after challenge (Figs. 4A, 4B, 4C and 4D); whereas, IL-17A-producing CD3^+^ CD4^+^ cells in immunized mice increased dramatically in response to GAS challenge (Fig. 4E). These results evidently indicate that the responsive IL-17A-producing cells are T helper cells and that SrtA/CTB immunization established memory Th17 cells that expanded in response to heterologous strains.

### 4. Clearance of GAS was promoted by T cells from immunized mice and prevented by depletion of IL-17A

**Figure 5:**
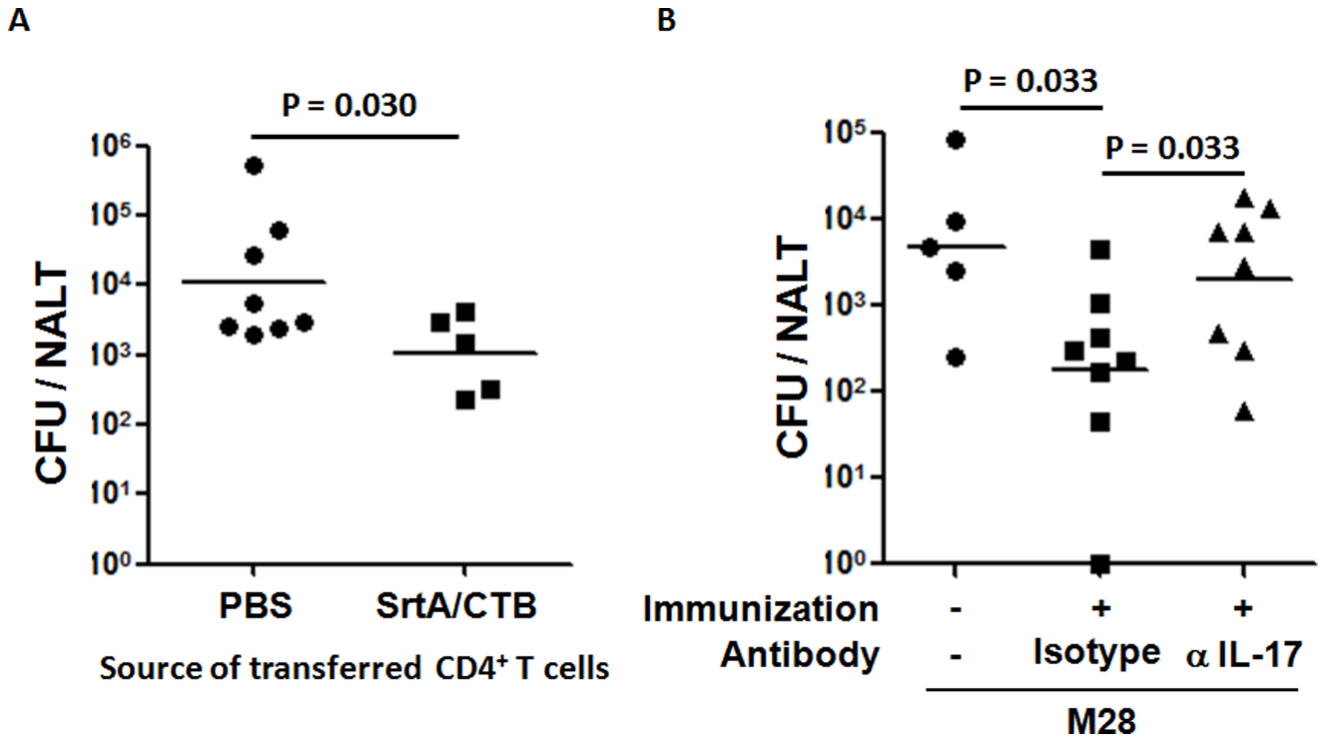
CD4^+^ IL-17A^+^ T cells promoted clearance of GAS from NALT. (*A*) C57B/6 mice were immunized with SrtA/CTB three times and euthanized 10 days later. Single-cell suspensions of lymph nodes and spleens were pooled and CD4^+^ T cells were purified then transferred to naïve WT C57B/6 mice. 24 hr after transfer recipients were challenged i.n. with M28 strain and euthanized 24 hr later for CFUs in NALT. (*B*) SrtA/CTB immunized mice received IL-17A neutralizing antibody or isotype control antibody i.p. several times prior to challenge with M28 strain. 24 hr after challenge mice were euthanized and NALT cell suspensions were plated for CFUs.

The next experiment questioned whether adaptive transfer of CD4^+^ T cells from SrtA/CTB-immunized mice, predominately populated with Th17 cells, could provide protection to naïve mice. Purified CD4^+^ T cells were isolated from SrtA/CTB-immunized mice and transfused into naïve mice by tail vein injection. 24 hr after cell transfer recipient mice were challenged i.n. with strain M28. The mice that received CD4^+^ T cells from immunized mice cleared streptococci more completely than those that received CD4^+^ T cells from naïve animals by 24 hr after challenge (Fig. 5A), indicating that CD4^+^ T cells provided protection. To further assess whether Th17 cells are the major contributors to the protection neutralizing antibody directed to IL-17A, mainly produced by activated Th17 cells, was administered to mice after SrtA/CTB immunization and prior to challenge with strain M28. SrtA/CTB-immunized mice that received isotype control antibody had lower numbers of CFUs in NALT relative to unimmunized mice. Conversely, much higher CFU numbers of M28 were recovered from mice that were received IL-17A neutralizing antibody (Fig. 5B), confirming that IL-17A plays an important role in clearance of streptococci from NALT.

### 5. SrtA-specific antibodies were dispensable for protection against GAS

**Figure 6:**
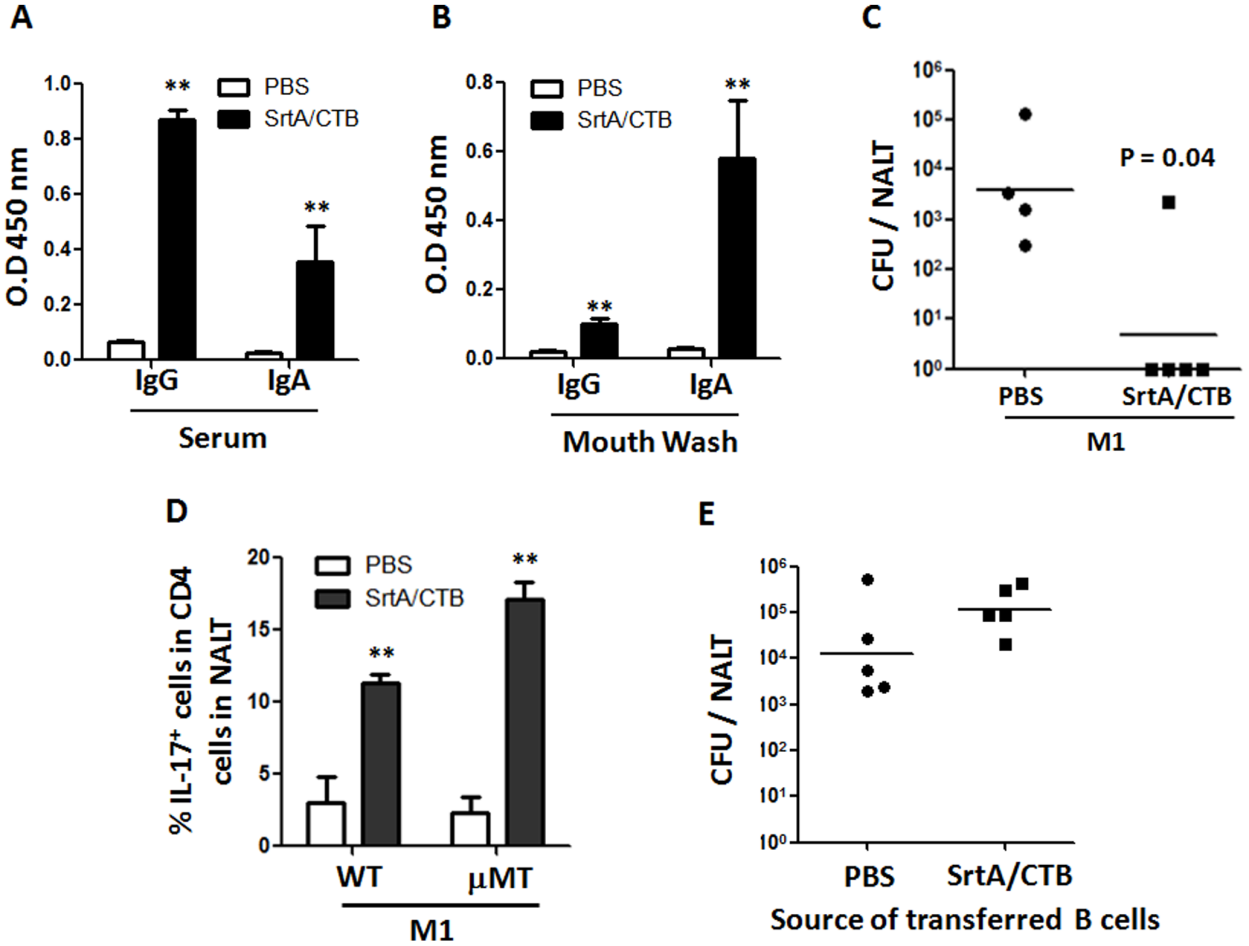
SrtA-specific antibodies were dispensable for protection. Mice (BALB/c) were immunized three times with SrtA/CTB and euthanized 10 days after the last immunization. Blood and oral wash were taken and samples of sera (IgG 1∶1000; IgA 1∶100) (*A*), and oral wash (neat) (*B*) were examined for antibody levels by ELISA (n = 6). (*C*) B cell deficient mice (µMT) were immunized with SrtA/CTB or received PBS and challenged as in Fig. 2, and euthanized 24 hr after challenge. NALTs were taken and homogenized for plating CFUs. (*D*) Quantification of CD4^+^ IL-17^+^ responses to M1 in NALT of WT and µMT mice before and after immunization. Data are the means ± SEM of three mice per group. ** *P*≤0.01. (*E*) C57B/6 mice were immunized as in Fig. 5A. Single-cell suspensions of lymph nodes and spleens were pooled and CD19^+^ B cells were purified then transfused to naïve WT C57B/6 mice. 24 hr after transfer recipients were challenged i.n. with M1 strain and euthanized 24 hr later for CFUs in NALT.

The above T cell adaptive transfer experiment suggested protection from i.n. challenge was independent of B cells and antibody. Samples of blood and oral washes were collected after immunization and SrtA-specific antibodies were measured by ELISA. Higher levels of IgG and IgA in samples of serum (Fig. 6A) and oral washes (Fig. 6B) were detected in immunized than in naïve mice. To further examine the role of antibodies in the protection, B cell-deficient mice (µMT) were vaccinated with SrtA/CTB. These mice produce no detectable B cells or antibodies (data not shown) but display normal development of the T lymphocyte compartment [25]. As predicted, after immunization µMT mice cleared the bacteria from NALT as efficiently as WT mice (Fig. 6C). Numbers of Th17 cells in NALT from µMT and wild type mice were comparable before and after immunized (Fig. 6D), suggesting that the protection in µMT mice is provided by T cells. This experiment didn't completely rule out a possible role of B cells in the protection because B cells in µMT mice are disabled. Therefore, B cells from immunized WT mice were transfused into naïve WT mice. 24 hr later these recipients were challenged with GAS. Similar numbers of CFU were found in the mice that received B cells from immunized (SrtA/CTB) and unimmunized mice (PBS) (Fig. 6E). Thus in this infection model immune clearance of streptococci is not depend on antibody.

### 6. Neutrophil influx was increased in NALT of SrtA/CTB-immunized mice in response to GAS challenge

**Figure 7:**
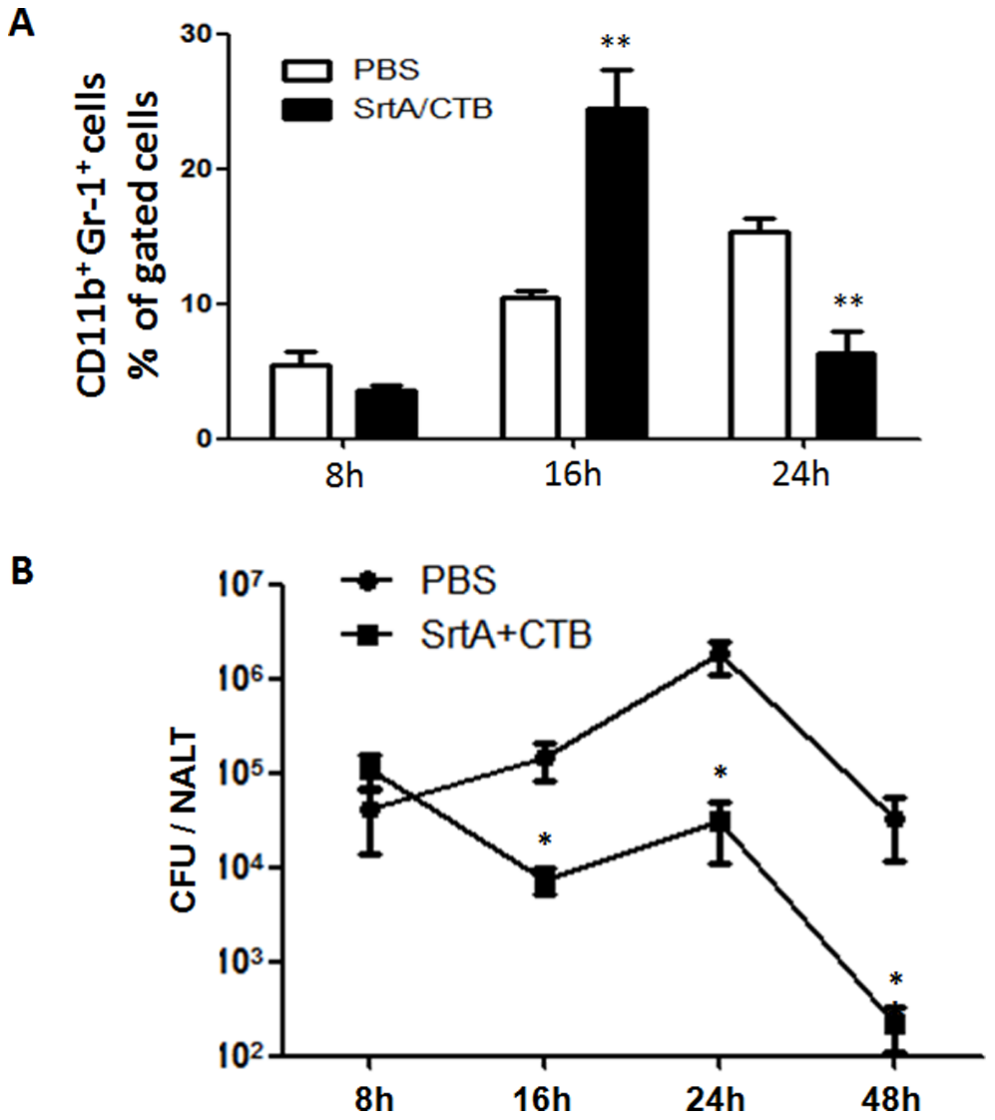
Neutrophil influx was rapidly increased in NALT of immunized mice in response to GAS challenge. Immunized C57B/6 mice were challenged with GAS M1 strain and sacrificed at the indicated time points after challenge. (*A*) Recruitment of neutrophils was determined by FACS analysis on NALT cells. Results are the mean ± SEM of three mice per group. (*B*) CFUs in NALT were measured by plating NALT homogenates on blood agar plates. Data are the means ± SEM of three mice per group. * *P*≤0.05, ** *P*≤0.01.

A neutrophil influx is a hall mark of GAS pharyngitis and these bacteria produce several factors that impede the phagocytic defenses [26]. IL-17 is known to promote neutrophil recruitment, activation, and efficient phagocytic killing [27]. The above experiments suggest that the robust Th17 response to multiple i.n. infections could recruit and activate neutrophils to the site of infections and that this leads to the more rapid and complete clearance of streptococci from NALT. Previous study demonstrates that infection of naïve mice with GAS induces an influx of neutrophils into NALT with a peak by 24 hr and diminished by 48 hr [19]. We postulated that neutrophil infiltration in NALT would be earlier or more robust in SrtA/CTB immunized mice. Flow cytometric analyses based on the differential neutrophil surface expression of CD11b and Gr-1 demonstrated more rapid influx of neutrophils into NALT, which peaked by 16 hr after GAS challenge in immunized mice (Fig. 7A). As anticipated CFUs were significantly reduced at this time point (Fig. 7B 16 hr) and were more completely cleared at later times (Fig. 7B 24 hr and 48 hr), compared with unimmunized mice. More neutrophils were found in PBS control mice than in immunized mice by 24 hr (Fig. 7A), but CFUs were significantly higher in these mice (Fig. 7B). These results suggest that the rapid neutrophil infiltration, enhanced by SrtA/CTB immunization is important for more efficient clearance of GAS.

## Discussion

Antigens that induce protection against a broad-spectrum of GAS serotypes and have a low risk of inducing autoimmunity are goals of vaccine development. Though many have been described, none have reached the stage of human trial. Incomplete understanding of immune protection against GAS infection has been a major obstacle to vaccine development. Early human vaccine studies showed that purified M protein vaccines induced protection to oral challenge with streptococci without induction of bactericidal antibody [3], [28]. Moreover, there are no human data to support the claim that serotype specific antibody is essential for prevention of pharyngitis or throat acquisition of streptococci; in fact the opposite is true. Wannamaker and colleagues found that men in the arm forces and members of families of an index case become streptococcal colonized and developed pharyngitis in the face of type specific bactericidal antibody [29]–[31]. Opsonic antibody is likely helpful to clear GAS from blood and sterile tissue, infections that are much rarer than pharyngitis. It was suggested from our previous report that immune protection induced by multiple i.n. infections was based on GAS specific Th17 cells [15]. The results presented here confirm that interpretation and demonstrate that multiple i.n. immunization with recombinant SrtA protein can induce Th17-based, antibody-independent mucosal immunity. These experiments also show that protection extends to multiple M types of GAS. These findings provide new understanding of protective mucosal T cell responses to GAS and offer an alternative strategy for development of an effective GAS vaccine.

As stated our experiments do not rule out a role for antibody in immune protection. Single pharyngeal infection of baboons results in antibody-dependent protection against homologous but not heterologous serotypes of GAS [32]. We reported that Th17 cells are not induced by a single but multiple live GAS immunization [15] and an efficient Th17 response was not achieved by a single immunization with SrtA/CTB (data not shown) but required multiple immunizations including CTB as adjuvant. We interpret this to mean that a high level of GAS specific memory Th17 are required and achieved by repeated inoculations with SrtA or GAS infections. This may explain why children usually experience frequent episodes of strep pharyngitis before incidence of diseases decreases at around 12 years old.

CD4^+^ IL-17^+^ cells can reach high numbers, up to 50% of CD4^+^ cells in NALT following multiple immunizations with live GAS [15]. However, only 15–20% of CD4^+^ T cells from NALT of B6 mice or 6–8% from BALB/c mice were IL-17^+^ cells following vaccination with SrtA/CTB. Still this moderate Th17 response efficiently cleared challenge bacteria in both strains of mice. This less vigorous response may lower the risk of complications associated with Th17 cells. Vaccination with SrtA is unlikely to induce autoimmune responses because SrtA is expressed by other Gram-positive bacteria that are not associated with autoimmune diseases.

Robust antibody responses were induced following SrtA/CTB immunizations. However, B cell-deficient mice were well protected after immunization and adoptive transfer of B cells from immunized mice did not promote GAS clearance in recipients. This might be due to inability of the antibodies to access SrtA because it is located inside the cell wall [33]. It was recently reported that antibodies against surface molecules of influenza virus fail to neutralize variant influenza strains, but CD4^+^ T cells that recognize internal conserved influenza proteins limit virus shedding and disease severity [34]. In addition, T cell biased and antibody-independent protection against *S. aureus* and *S. pneumoniae* was also reported [35], [36]. The SrtA-induced protective Th17 immunity suggests that conserved non-surface bacterial antigens with T cell epitopes can be used as a new class of vaccine candidate to cope with microbial antigen variation.

Avoiding phagocytic eradication by multiple mechanisms is a major virulence feature of GAS [26]. Neutrophil influx in NALT was delayed and eradication of GAS was inefficient in unimmunized mice; however, neutrophils rapidly increased in NALT of immunized mice and bacterial cells were more efficiently cleared. We postulate that activated neutrophils by adaptive T cell response bypass GAS antiphagocytic virulence, leading to enhanced efficiency of bacterial clearance. Therefore, activated neutrophil phagocytic defense plays a critical role in anti-GAS immunity and could be targeted for prevention of GAS infections. Future studies will be required to determine the levels of Th17 cells specific to SrtA and other conserved GAS molecules in humans and the role of these cells in resistance to streptococcal infection.
